# At the heart of organ donation. Case reports of organ donation after cardiac death in two patients with successfully repaired AAST grade V cardiac injuries

**DOI:** 10.1186/s13017-019-0279-5

**Published:** 2019-12-19

**Authors:** Paola Fugazzola, Luca Ansaloni, Marco Benni, Alessandro Circelli, Federico Coccolini, Emiliano Gamberini, Andrea Nanni, Emanuele Russo, Matteo Tomasoni, Vanni Agnoletti

**Affiliations:** 1 Unit of Emergency and General Surgery, Bufalini Hospital, Viale Ghirotti 286, 47521 Cesena (FC), Italy; 2 Intensive Care Unit, Bufalini Hospital, Viale Ghirotti 286, Cesena, Italy; 3 General, Emergency and Trauma Surgery Department, Pisa University Hospital, Pisa, Italy

**Keywords:** Heart injury, Organ donation, Extracorporeal membrane oxygenation (ECMO), Donation after circulatory death (DCD)

## Abstract

**Background:**

Trauma victims could be an important source of organs. This article presents two cases of successful organ donation and transplant, after Maastricht category III cardiac death in patients with successfully repaired AAST grade V traumatic cardiac injuries.

**Case presentation:**

The first donor was an adult patient with self-inflicted heart stab wound and non-survivable burn injury. The second one was an adult patient with blunt cardiac and abdominal trauma and an anoxic brain injury due to a car accident. The cardiac injury was promptly repaired in both patients. In the first case, adequate organ perfusion ante-mortem was achieved thanks to venoarterial extracorporeal membrane oxygenation and intensive care unit support. The above procedure allowed successful organ donation and transplantation even after Maastricht category III cardiac death. This is the first case reported where, for organ donation purposes, it was made necessary first thing to avoid the immediate death of the patient, due to a rare and frequently not survivable cardiac injury. The challenge of preserving organ perfusion, due to major burn injury effects, was faced afterwards.

**Conclusions:**

The outcomes of these two cases suggest that a repaired heart injury should not be considered as an absolute contraindication to organ donation, even if it is associated with non-survivable major burns. Therefore, cardiac death could provide an opportunity for these kinds of patients to contribute to the pool of potential organ donors.

## Background

Trauma victims are an important source of organs. A recent review based on the US Scientific Registry of Transplant Recipients showed that trauma donors generally produce more organs and better kidneys per donor, than non-trauma donors. Furthermore, trauma donors are an extremely important source for all extrarenal organs, aside from the liver [1]. The reason could be that trauma donors are younger and healthier than their non-trauma counterparts. In addition to that, over the past three decades, the improvements in trauma systems, resuscitative methods, and ICU level care might have led to a decrease in multiorgan failure prior to death [1].

The great majority (84%) of trauma donors are head trauma patients [1] with neurologic determination of death. However, in response to an increased demand for organ procurement, non-heart beating or Maastricht category III cardiac death (DCD) have recently re-emerged as possible cases to expand the potential donor pool. It poses ethical challenges to include patients with fatal non-neurological conditions within the potential donor pool, especially in the context of end-of-life care, and medical problems. The challenges are set by severity of hypoperfusion and ischemic organ injury, as these two remain the dominant factors in influencing graft outcomes in these patients [2]. The proportion of DCD has increased in both trauma and non-trauma donors from less than 5% in 2000 to over 15% in 2016 [1].

Major burns have traditionally been considered a contraindication to organ donation. This is due to possible risks of splanchnic ischemic injury after burn shock, together with high risks of bacterial contamination and sepsis in burnt patients [2–6].

**Fig. 1:**
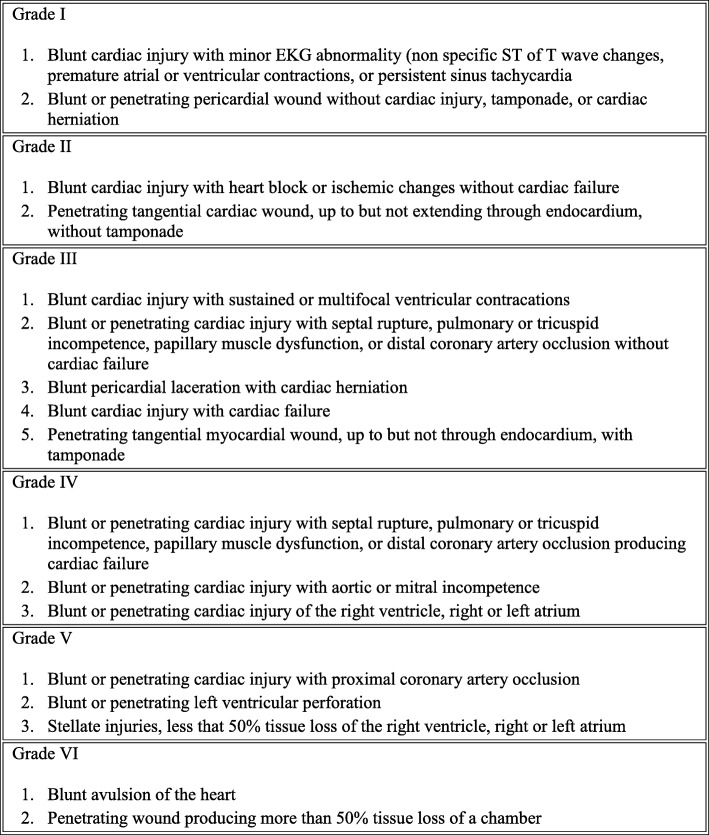
AAST Injury Scale: cardiac injuries

Here below, we present two cases of successful organ donations and transplantations after Maastricht category III DCD, with successfully repaired AAST grade V traumatic cardiac injuries (Fig. 1). The first donator was an adult patient with self-inflicted heart stab wound and non-survivable burn injury; the second one was an adult patient with blunt cardiac and abdominal trauma after a car accident and an anoxic brain injury.

## Cases presentation

### Case 1

A 30-year-old male was admitted to the Emergency Department for a penetrating self-inflicted left chest stab wound, extensive (80% total body surface area, TBSA) full-thickness burn injury, and carbonization. During the pre-hospital phase, the patient was intubated and the initial resuscitation was performed through crystalloids infusion. He was transferred to the nearest “hub” Trauma Center by air ambulance. At the arrival, he was hypotensive (systolic blood pressure (SBP) 80 mmHg) and tachycardic (heart rate (HR) 150 bpm). The stab wound was in the left third intercostal space, medial to the midclavicular line. The chest x-ray showed a left hypertensive massive hemopneumothorax. An E-FAST was performed, but in the subcostal window, the pericardium was not assessable, probably because of the acoustic barrier caused by skin carbonization. A left minithoracotomy was performed and a chest drainage was put in place; this was followed by the immediate return of 3000 ml of blood and air. A thromboelastography (ROTEM) and an arterial blood gas test (ABG) were performed: pH 6.8, base excess (BE) − 22, lactates 14. Tranexamic acid 1 g, two units of red blood cells, and two plasma units were transfused. The patient was transferred to the operating room (OR) to perform a thoracotomy. During the resuscitation phase, the specialist carried out a burn evaluation. A very bad prognosis was determined due to the severity of the burn injuries.

In the OR, a clamshell incision was performed and a pericardial lesion was found. A pericardiotomy showed a left ventricular full-thickness injury (grade V according to OIS-AAST system). After placing a Foley catheter in the cardiac wound, a direct prolene and metal staples suture were performed. The Foley catheter was removed without residual bleeding. Bilateral chest drainages were put in place and the thoracic wall was closed (Additional file 1). A bilateral lower limb escharotomy was performed. After the procedures, the SBP was 120 mmHg, the HR 120 bpm, and the ABG test showed the following results: pH 7.28, BE − 8.9, lactates 12, Hb 8.9 mg/dL. Thereafter, it was applied a goal directed therapy of the coagulopathy according to ROTEM results.

The patient received continuous support in intensive care unit. Due to hemodynamic instability, venoarterial extracorporeal membrane oxygenation (V-A ECMO) was initiated. This procedure allowed to preserve organs, to allow appropriate family consultation and palliative care planning. During family discussions regarding end-of-life care, the feasibility of organ donation was raised.

The further resuscitation (Table 1) allowed confirmation of medical suitability (Table 2).

**Table 1:** Interventions during the donor management in intensive care unit (ICU)

	Patient 1	Patient 2
Time of management in ICU	19 h	16 days
TBSA burn %	> 80%	0%
Weight (kg)	70	109
Antibiotic therapy	Amoxicillin/Clavulanic acid	Cefazoline, Gentamicin, Piperacillin/Tazobactam, Amikacin, Clindamycin
Plasma (mL)	2785	0
EC (units)	5	1
Crystalloids (ml)	In the first 19 h from ICU arrival: 5500	In the first 24 h from ICU arrival: 3500
Corticosteroids	Hydrocortisone 750 mg	Hydrocortisone 240 mg/die
Sedation/analgesia	Sufentanil/Midazolam/Ketamine	Sufentanil/Ketamine/Methadone/Propofol/Paracetamol/Diclofenac
Inotropes	Noradrenaline/Adrenaline	Noradrenaline/Dobutamine
Enteral nutrition (ml)	380	0

**Table 2:** Clinical status of the donor

	Patient 1		Patient 2	
	At the arrival in the ICU	Prior to WCRS	At the arrival in the ICU	Prior to WCRS
O2 saturation (%)	100	96	100	100
End tidal CO_2_ (mmHg)	34	29	34	37
pH art	7.26	7.22	7.22	7.47
PaCO_2_ (mmHg)	37	43.2	42.4	34.6
PaO_2_ (mmHg)	106	520.8	147.4	140.1
HCO_3_ art (mMol/l)	16.6	16.7	16.3	25.7
BE art	− 10	− 9.4	− 10.2	1.4
Lactates art (mMol/L)	7.55	11.4	3.74	0.95
PaO_2_/FiO_2_	274	633	368	467
PvcCO_2_ (mmHg)	54.6	44.7	55.1	42.9
PvcO_2_ (mmHg)	41.4	27.1	44.1	40.2
SvcO_2_ (%)	72.4	45	74.9	70.5
Hb (g/dL)	14.7	7	13.8	8.1
Temp (°C)	32.7	32.7	34.5	37.8
SBP (mmHg)	84	99	121	115
DBP (mmHg)	52	58	72	55
HR (bpm)	135	98	85	74
Urine output (ml/h)	17	20	80	90

*ICU* intensive care unit, *WCRS* withdrawal of cardio-respiratory support, *PaCO*_*2*_ partial pressure of oxygen in the arterial blood, *PaO*_*2*_ partial pressure of CO_2_ in the arterial blood, *art* arterial blood, *BE* base excess, *FiO*_*2*_ fraction of inspired oxygen, *Hb* hemoglobin, *Temp* temperature, *SBP* systolic blood pressure, *DBP* diastolic blood pressure, *HR* heart rate

Death ascertainment took place 23 h after the arrival of the patient at the ED. The necessary procedures for DCD process for therapeutic transplantation purposes started only after the ascertainment of death with cardio-circulatory criteria and family approval.

Medical suitability for liver and kidneys donation was assessed by the Regional Reference Center for Transplants. Normothermic regional perfusion was started according to the standard procedure [7]. After the reperfusion phase, the liver was considered unusable due to ischemic injury. One kidney was not transplanted because of technical problems. However, one kidney was successfully transplanted.

### Case 2

A 47-year-old female was admitted to the Emergency Department for a blunt chest and abdominal trauma. Her car crashed against a bus near the Trauma Center. Her body was extricated with difficulties. During the pre-hospital phase, the patient had GCS 7, not detectable SpO2, and evident signs of hemorrhagic shock. She was quickly transferred to Trauma Center. At the arrival, she had a clear hemorrhagic shock. It was administered a rapid-sequence induction for emergency endotracheal intubation with Ketamine 100 mg and Succinylcholine 100 mg. A bilateral minithoracotomy was performed, but there was a rapid evolution in pulseless electrical activity (PEA). Chest X-ray showed an upper mediastinal widening and multiple broken rib fractures. The pelvis X-ray was negative. An E-FAST showed cardiac tamponade and fluid in the right abdomen upper quadrant. It was administered 1 mg of Adrenalin, and a resuscitative thoracotomy with pericardiotomy was performed together with circle restoration. A ROTEM and an ABG were carried out. Tranexamic acid 1 g, two units of red blood cells, and Fibrinogen 2 g were infused. Furthermore, she reported a right knee exposed fracture. The patient was transferred to the OR.

In OR, a clamshell incision was performed, and a blast full-thickness left auricle injury (grade V according to OIS-AAST system) was found (Additional file 2). A direct prolene suture was carried out. Internal cardiac massage and defibrillation (30 J) was required for rhythm restoration, due to onset of ventricular fibrillation. Sodium bicarbonate 8.4% 200 mL, calcium chloride 3 g, magnesium sulfate 1 g, and Amiodarone 300 mg were infused. It was also given noradrenaline infusion with target SBP 110 mmHg. After fluid resuscitation, due to the sudden appearance of abdominal distension, an urgent laparotomy with evidence of hepatic laceration and an abdominal packing was executed. A panaortography was carried out in the OR; it ruled out active bleeding. Having reached a partial hemodynamic stabilization, a temporary thoracic and abdominal closure was performed. During the surgical intervention, six units of red blood cells, two units of fresh frozen plasma, one unit of platelets, and Fibrinogen 1 g were infused. The patient was transferred to the Radiological Department to receive a total-body CT scan and, after excluding other immediately life-threating injuries, to the ICU.

The patient received continuous support in ICU (Table 1). Rapid hemodynamic stabilization and gradual improvements in respiratory exchanges took place. Due to the onset of acute kidney injury, CVVHDF was initiated. Seventy-two hours after the trauma, the patient underwent a surgical intervention of packing removal, definitive abdominal closure, pericardium plastic with porcine biological prosthesis (leaving an open upper window), and costal stabilization. Five days after trauma, the first neurologic window was made with a GCS of 6. A percutaneous tracheostomy was performed. Thirteen days after trauma, the following was observed: GCS 3t, myotic, isochoric, and non-reactive pupils, hypertonic lower limbs. An electroencephalogram and a brain MRI scan showed a diffuse hypoxic-ischemic damage. Sixteen days after trauma, the patient showed persistent GCS 3t, non-reactive pupils, presence of respiratory trigger, carinal reflex, and diffuse flaccidity. All the necessary neurological assessments have been performed in order to formulate a correct prognosis from the neurological point of view.

In light of the poor prognosis and after appropriate family consultation, a palliative care plan was initiated. During family discussion regarding end-of-life care, the feasibility of organ donation was raised.

Table 2 reports the clinical status of the patients before withdrawal of cardio-respiratory support.

The ascertainment of death took place 16 days after the arrival of the patient at the ED. After death ascertainment with cardio-circulatory criteria and following the non-opposition from the family, the necessary procedures for DCD process for therapeutic transplantation purposes were started.

The determination of medical suitability for the donation of the liver, lungs, kidneys, skin, and corneas was assessed by the Regional Reference Center for Transplants. Normothermic regional perfusion was started by positioning two femoral cannulas (venous and arterial) and aortic balloon in the contralateral femoral artery, according to the standard procedure [7]. The lungs were considered unusable due to the traumatic contusions. The liver and kidneys were successfully transplanted.

## Discussion and conclusions

Cardiac trauma is one of the most lethal injuries. Autopsy reports suggest that severe cardiac injury may carry a prehospital mortality as high as 95% [8]. Mortality among those surviving to ED evaluation remains high, estimated at approximately 80% [9], and surgical intervention is associated with poor outcomes for atrial or ventricular injuries with mortality between 40 and 70% [8]. Mortality is higher in cases of left ventricular injury [10]. This article reports two cases of successful management of severe cardiac trauma. In both cases, the patient died due to the associated lesions (non-survivable burn injury in case 1 and anoxic brain injury in case 2). However, the surgical treatment associated with the optimal ICU management allowed to maintain an adequate organ perfusion ante-mortem which has, in turn, allowed the procurement of transplantable organs.

Current literature on organ retrieval in donors with severe burns is limited. It mainly relates to brain-dead donors (DBD), who died as a result of associated anoxic brain injury [3–6]. Widdicombe et al. reported two successful cases of organ donation and transplantation after Maastricht category III cardiac death, in adult patients with non-survivable burn injuries. Good outcomes were achieved for both cases [2]. DCD in burn injured patients raises medical challenges related to the potential risks of splanchnic ischemic injury following burn shock, together with a high risk of bacterial contamination and sepsis [2–6].

Furthermore, DCD poses some ethical challenges, as it directly affects the timing of withdrawal of cardio-respiratory support (WCRS). This is mainly due to donor evaluation requirements, potential recipient identification, and general logistics, in order to coordinate surgical and theater resources (2). For a DBD donor with non-survivable burns, interventional support is maintained after declaration of death, in order to enable donor evaluation and donation feasibility. While, for DCD donors, interventional support is required ante-mortem.

In the past, the risk of ischemic organ injury, caused by burn shocks, was the rationale for considering major burns as a contraindication to organ donation. With acute intensive care progresses and innovations, this contraindication has lost absoluteness [2, 6]. As a matter of fact, the reported organ survival rates for major burned donors reached 86% [5] and the long-term outcomes are good too [4] (3). However, there is no published literature advising on early resuscitation in patients with non-survivable burns to facilitate organ donation, including fluid formulae and fluid type, use of adjuncts such as inotropes, optimal monitoring, and clinical endpoints. Moreover, there are no clinical nor laboratory indices able to determine a donor’s suitability in burns [2]. Furthermore, no clear indications are given about an optimal timeframe to retrieve organs from fatal burns donors, in order to reduce the development of severe systemic inflammatory response syndrome [2].

In our cases the V-A ECMO, together with the optimization of circulating volume during the 19 h in the ICU, allowed to maintain an adequate organ perfusion ante-mortem which has, in turn, allowed the procurement of a transplantable kidney.

Case 1 is the first reported where, even before facing the challenge of preserving organ perfusion from major burn injury effects, it was made necessary for organ donation purposes, to avoid the immediate death of the patient for a rare and frequently not survivable cardiac injury.

In case 1, the pericardial hemorrhage drained into the left pleural cavity, and resulted in a hemothorax without a cardiac tamponade. Clinical signs of cardiac tamponade were not present, and it was impossible to perform a US to evaluate the pericardium, because of the acoustic barrier caused by the carbonization of the skin. Therefore, this scenario prevented us from formulating a diagnosis of cardiac injury, before bringing the patient to the operating room.

In case 2, a cardiac tamponade was found.

It is a matter of debate whether the development of pericardial tamponade is a protective factor in cardiac injuries [10–12]. According to some authors, in the absence of pericardial tamponade, fatality rate is higher, as a diagnosis of heart injury can be more challenging [12]. In other studies, the presence of pericardial tamponade is a critical independent predictive factor for mortality in a multivariate analysis [10].

The outcomes of these cases suggest that trauma donors have a vital role in meeting organ demands. Consequently, trauma surgeons and intensivists, in particular, should consider the potential for organ donations, while evaluating and resuscitating even the most gravely injured patients. In this context, repaired heart injuries, even associated with non-survivable major burns, should not be considered as an absolute contraindication to organ donation. Furthermore, cardiac death provides opportunity for these kinds of patients, to contribute to the pool of potential organ donors.

## Supplementary information

**Additional file 1.** Patient 1: video of surgical operation.

**Additional file 2.** Patient 2: video of surgical operation.

## Data Availability

Not applicable

## Abbreviations

AAST: American Association for the Surgery of Trauma
ABG: Arterial blood gas test
BE: Base excess
CVVHDF: Continuous venovenous hemodiafiltration
DBD: Donors after brain death
DCD: Donation after cardiac death
E-FAST: Extended focused assessment with sonography for trauma
GCS: Glasgow Coma Scale
HR: Heart rate
ICU: Intensive care unit
OR: Operating room
PEA: Pulseless electrical activity
ROTEM: Rotational thromboelastometry
SBP: Systolic blood pressure
TBSA: Total body surface area
V-A ECMO: Venoarterial extracorporeal membrane oxygenation
WCRS: Withdrawal of cardio-respiratory support
